# The Precursor for Nerve Growth Factor (proNGF) in Thyroid Cancer Lymph Node Metastases: Correlation with Primary Tumour and Pathological Variables

**DOI:** 10.3390/ijms20235924

**Published:** 2019-11-25

**Authors:** Christopher W. Rowe, Tony Dill, Sam Faulkner, Craig Gedye, Jonathan W. Paul, Jorge M. Tolosa, Mark Jones, Simon King, Roger Smith, Hubert Hondermarck

**Affiliations:** 1 School of Medicine and Public Health, University of Newcastle, Callaghan 2308, Australia; 2 Department of Endocrinology, John Hunter Hospital, Newcastle 2310, Australia; 3 Hunter Medical Research Institute, 1 Kookaburra Circuit, New Lambton Heights 2310, Australia; 4 Department of Anatomical Pathology, NSW Health Pathology (Hunter), Newcastle 2310, Australia; 5 School of Biomedical Sciences and Pharmacy, University of Newcastle, Callaghan 2308, Australia; 6 Department of Medical Oncology, Calvary Mater Newcastle, Waratah 2298, Australia

**Keywords:** thyroid cancer, proNGF, neurotrophin, metastasis, nerves

## Abstract

Metastases in thyroid cancer are associated with aggressive disease and increased patient morbidity, but the factors driving metastatic progression are unclear. The precursor for nerve growth factor (proNGF) is increased in primary thyroid cancers, but its expression or significance in metastases is not known. In this study, we analysed the expression of proNGF in a retrospective cohort of thyroid cancer lymph node metastases (*n* = 56), linked with corresponding primary tumours, by automated immunohistochemistry and digital quantification. Potential associations of proNGF immunostaining with clinical and pathological parameters were investigated. ProNGF staining intensity (defined by the median h-score) was significantly higher in lymph node metastases (h-score 94, interquartile range (IQR) 50–147) than in corresponding primary tumours (57, IQR 42–84) (*p* = 0.002). There was a correlation between proNGF expression in primary tumours and corresponding metastases, where there was a 0.68 (95% CI 0 to 1.2) increase in metastatic tumour h-score for each unit increase in the primary tumour h-score. However, larger tumours (both primary and metastatic) had lower proNGF expression. In a multivariate model, proNGF expression in nodal metastases was negatively correlated with lateral neck disease and being male. In conclusion, ProNGF is expressed in locoregional metastases of thyroid cancer and is higher in lymph node metastases than in primary tumours, but is not associated with high-risk clinical features.

## 1. Introduction

Thyroid cancer is the most common endocrine malignancy, with rising incidence [1]. Differentiated thyroid cancers arise from the thyroid follicular epithelium, and develop in either papillary or follicular growth patterns. The majority of differentiated thyroid cancers are localised within the thyroid in the early stages, and have an excellent prognosis if removed prior to the development of metastases, with >95% 10-year survival [2]. However, approximately 20% of thyroid cancers have metastases at diagnosis, usually loco-regionally within the lymph nodes of the neck or, less commonly, at distant sites such as lung and bone, and represent a more aggressive subset, with increased morbidity and mortality [2,3]. However, the size and location of metastases appears to be important in prognosis. Microscopic deposits of cancer within the lymph nodes of the central compartment of the neck appear to have little prognostic significance, whilst larger nodal involvement, and the involvement of lymph nodes in the lateral compartment of the neck, increase the risk of local recurrence following treatment and are associated with reduced overall survival [4,5].

The precursor for nerve growth factor (proNGF) is a soluble protein of 246 amino acids, transcribed from the *NGF* gene on chromosome 1p13. ProNGF and NGF are involved in the survival, growth and differentiation of neurons in the central and peripheral nervous system [6]. Interestingly, increasing evidence reports the role of proNGF and NGF in stimulating cancer progression [7,8,9,10]. In thyroid cancer, ProNGF has been shown to be overexpressed in differentiated cancers of both papillary and follicular subtypes [11]. Based on prior data, we hypothesised that proNGF would be expressed in the metastases of thyroid cancer, and that expression of proNGF would correlate with tumour aggressiveness, which could suggest a role for proNGF as a prognostic biomarker for thyroid cancer aggressiveness, or may suggest a mechanistic target for future research.

Therefore, we designed and conducted a study to characterise the expression of proNGF in clinical specimens of thyroid cancer lymph node metastases, linked with analyses of the primary tumour, and correlated with clinical and pathological characteristics. The data show that proNGF is overexpressed in nodal metastases of thyroid cancers compared to corresponding primary tumours, but we found no correlation between proNGF expression in lymph node metastases and cancer aggressiveness. 

## 2. Results

ProNGF expression was analysed by immunohistochemistry and digital quantification in a total of 112 whole-slide tissue sections from patients with thyroid cancer, corresponding to 56 lymph node metastases of thyroid cancer paired with primary tumours from the same patients. ProNGF intensity is presented as an h-score (a numeric scale between 0 and 300, with higher numbers representing greater immunostaining and protein expression). Demographic and clinical information for the included samples are presented in Table 1.

### 2.1. Validation of Automated Scoring Algorithm (h-score)

**Figure 1:**
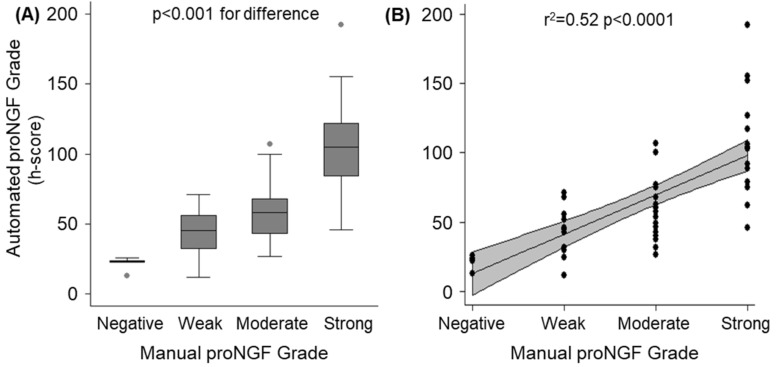
Validation of automated immunohistochemistry analysis (h-scores) compared to manual grading for the cohort of 56 primary tumours. (**A**): Box and whisker plot demonstrating distribution of automated h-scores, stratified by manual scores. Significant between-group differences were confirmed by ANOVA (*p* < 0.001). (**B**): Scatter-plot of individual automated h-scores (dots) over manual grading. The solid line represents the regression fit, with the grey band demonstrating the 95% CI of the regression line (r^2^ = 0.52, *p* < 0.0001).

Prior to proceeding with the analysis, the performance of the h-score (automated digital analysis) was confirmed by correlating with manual grading. Two operators who were blinded to the h-score independently assigned a manual score of proNGF immunhistochemical intensity of staining on a four-point scale (negative/weak/moderate/strong) on the full cohort of primary tumours (*n* = 56). The results are presented in Figure 1. Overall, there was an acceptable correlation between the manually assigned scores and the automated h-scores (r^2^ = 0.52, *p* < 0.0001). The median h-scores for the five cases manually classified as “0: negative” were 23 (interquartile range 22–24).

### 2.2. ProNGF Expression in Thyroid Cancer

**Figure 2:**
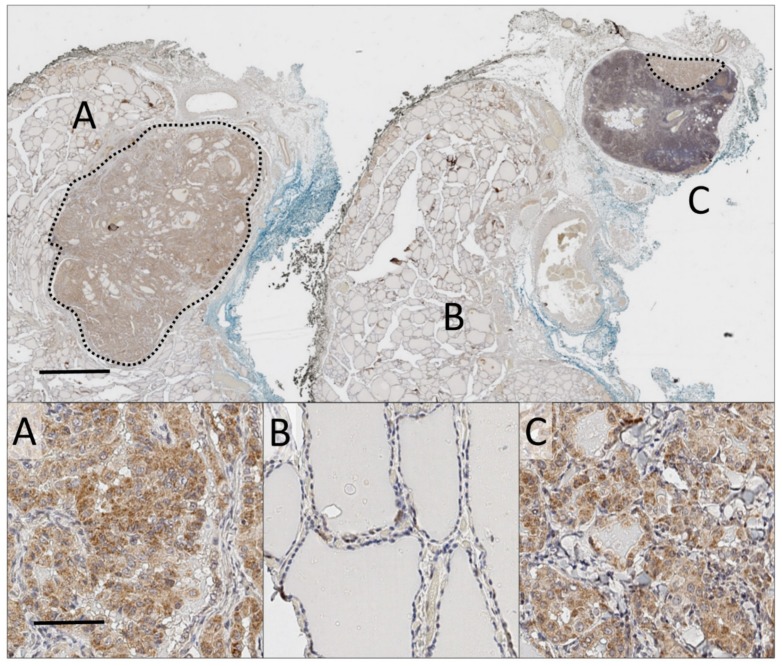
Whole-slide analysis of a primary thyroid cancer and corresponding thyroid cancer nodal metastasis. Main: Low magnification (0.5×) of whole-slide of proNGF immunostaining (DAB, brown), with haematoxylin nuclear counterstain (blue), of primary tumour and paired metastasis. Blue and black inking represent the anterior and tracheal borders of thyroid respectively. Scale bar 2mm. (**A**) 7 mm microPTC with staining for proNGF. (**B**) Adjacent normal thyroid tissue. (**C**) 2 mm lymph node micro-metastasis at upper edge of node, included in same anatomical block. Dotted lines demarcate tumour. Inset: 20× magnification of representative fields. Scale bar 50 µm.

**Figure 3:**
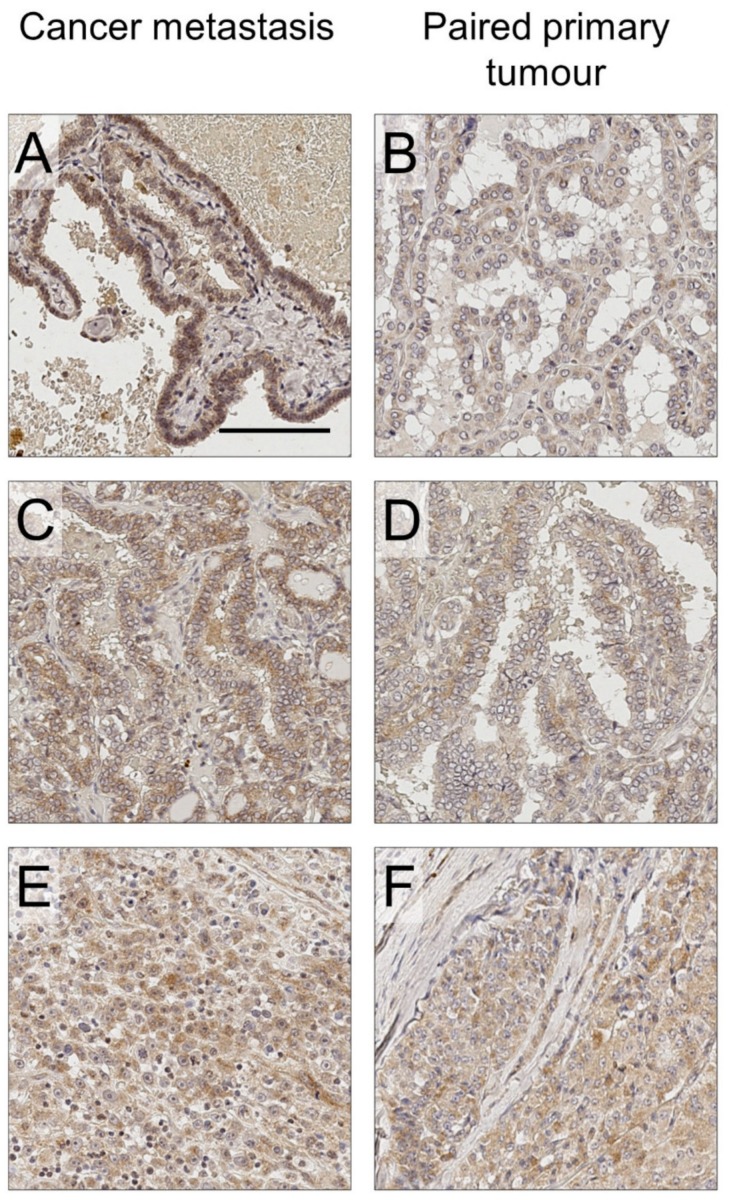
ProNGF immunostaining of thyroid cancer lymph node metastasis, compared with paired primary tumour. (**A**) Papillary thyroid cancer metastasis, h-score 148. (**B**) Primary tumour of A, h-score 56. (**C**) Papillary thyroid cancer metastasis, h-score 154. (**D**) Primary tumour of C, h-score 71. (**E**) Follicular thyroid cancer metastasis, h-score 60. (**F**) Primary tumour of F, h-score 60. Scale bar: 100 µm. 20× magnification.

ProNGF expression was detected in the majority of thyroid cancers and lymph node metastases (Table 1). It was found that 93% of sections from the papillary subtype and 75% from the follicular subtype expressed proNGF (positive expression was classified as h-score greater than 25, see Figure 1a for justification). An example of proNGF immunostaining is presented in Figure 2, showing a primary thyroid cancer and an adjacent lymph node metastasis, both expressing proNGF. ProNGF immunostaining was in the cytoplasm of cancer cells (Figure 2 and Figure 3), which is the expected location for this protein to be expressed. Minimal staining of fibroblasts and adjacent stroma was noted (Figure 3a,f). Some specimens contained adjacent benign thyroid follicular cells, which generally had low expression of proNGF (Figure 2b). However, due to nonspecific uptake of DAB staining by colloid in the benign regions, the h-scores were not relaible. 

The majority of primary tumours demonstrated uniform proNGF expression throughout the tumour; however, 25% showed a predominantly peripheral pattern of staining around the anatomical edge of the tumour (for example, Appendix B, Case 3). Peripheral immunostaining appeared to correlate with the biological properties of the tumour, as it was associated with the anatomical edge of the tumour rather than the cut edge of the pathology specimen, and was not caused by increased cellular density. Peripheral pattern staining did not correlate with the clinical or pathological parameters of the tumour.

**Figure 4:**
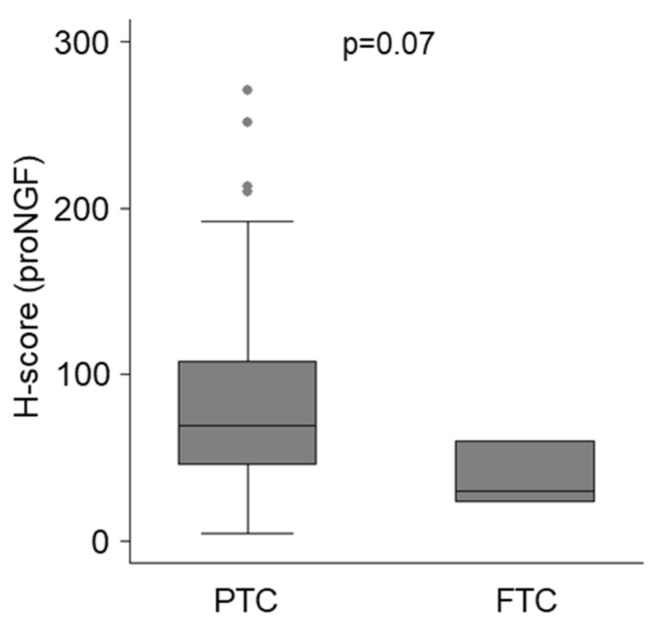
ProNGF immunostaining of thyroid cancer, stratified by histopathological subtype. Data for primary and metastatic lesions for PTC (*n* = 106) and FTC (*n* = 4) are combined. For analysis sub stratified by histopathological subtype, and by location of lesion, see Table 1.

Considering all neoplastic tissues together (pooled analysis of primary and metastatic samples), papillary thyroid cancer had a trend towards higher expression of proNGF (Figure 4, median h-score 70, IQR 46–108) than follicular thyroid cancer (median h-score 30, IQR 23–60, *p* = 0.07 for difference). Within papillary thyroid cancers, there was no difference in proNGF expression between tumours expressing a classical architecture, as compared to those with follicular-variant histology (Table 1). One paired case of anaplastic thyroid cancer had low proNGF expression. 

### 2.3. ProNGF Expression in Lymph Node Metastases Positively Correlates with Primary Tumours

**Figure 5:**
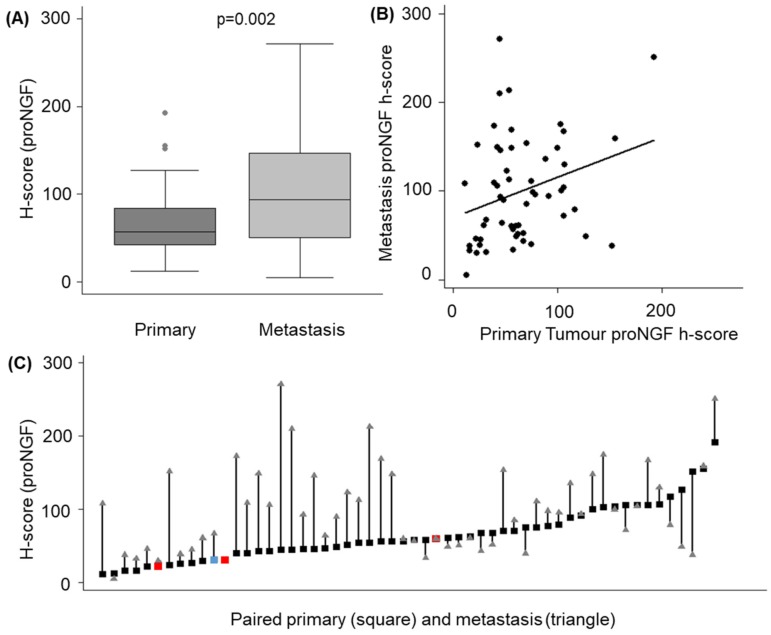
Quantification of proNGF staining in primary thyroid tumours vs lymph node metastases. (**A**) H-score for proNGF intensity in primary tumours and linked metastases. (**B**) Scatter plot showing correlation between h-score in primary and paired metastases (**C**) Individual data points for the 56 paired cases of primary tumour (squares, PTC = black, FTC = red, ATC = blue) and nodal metastases (triangle).

Median proNGF h-scores were higher in the nodal metastases (94, IQR 50–147), compared to the matched primary specimens (57, IQR 42–84, *p* = 0.002) (Table 1, Figure 5a). Quantile regression of metastatic h-scores on primary h-scores adjusted for age (≥55 years) and sex (male) confirmed this relationship, with an increase of 0.68 units in the median metastatic h-score for each unit increase in the primary score (95% CI 0 to 1.2, *p*-value = 0.009, Figure 5B). This pattern continued to be observed following a sensitivity analysis removing two outliers (median metastatic h-score increased 0.58 units (95% CI 0 to 0.9, *p* = 0.05) for each unit increase in the primary score). Finally, of the 56 metastases, 39 (69%) had higher expression of proNGF than the primary tumour (Figure 5c). 

### 2.4. ProNGF Expression is not Associated with High Risk Clinical Features

A univariate analysis comparing proNGF expression to clinical and pathological variables was undertaken separately for primary tumours and metastases (Table 1). ProNGF expression in the primary tumour was inversely associated with tumour size and TNM stage (*p* < 0.05). In metastases, proNGF expression was inversely associated with the size of the involved lymph node, and with the location of the lymph node in the lateral neck. We then constructed an exploratory multivariate model to assess whether the characteristics of the primary tumours were associated with proNGF expression, which included covariates of age and gender (base model), and tumour size (≥4 cm), the presence of vascular invasion, the presence of extra-thyroidal extension and multifocality in the full model. The results are presented in Table 2. In the full model, the parameter estimate for the primary tumour h-score was similar to that of the model adjusting for age and gender alone, suggesting no evidence of confounding from any of the model variables (Table 2A). In the full model, none of the covariates were significantly associated with the h-score in the primary tumour. 

A separate multivariate model was fit examining lymph node metastases, and including variables of covariates for age (≥55 years), sex, metastases size (≥2 mm), location (lateral neck) and timing of detection (>6 months from primary); it identified significant effects for sex and neck site (Table 2B). Specifically, males had lower median proNGF h-scores (−35, 95% CI −60 to −10, *p*-value = 0.008), and the presence of lateral neck disease was associated with a −47 (95% CI −74 to −20, *p*-value < 0.001) change in the median proNGF h-score.

### 2.5. ProNGF Expression in Primary Tumours does not Predict Metastases

To determine whether proNGF expression in primary tumours was associated with metastatic potential, we compared the h-scores of primary tumours with metastases to a cohort of primary tumours without lymph node metastases (*n* = 29, comprising 18 PTC and 11 FTC, with mean age 57 years, 59% female and mean tumour size 25 mm). The median h-score in primary tumours without nodal metastases was 45 (IQR 26–68), compared to 57 (42–84) in tumours with metastases (*p* = 0.16). Similar results were obtained when comparing the PTC subgroup alone. Using a multiple logistic regression model to examine for potential confounding, with the presence or absence of locoregional metastases as the dependent variable, and including model variables of primary tumour proNGF h-score, age, gender, tumour size, the presence of extra-thyroidal extension and the presence of vascular invasion, proNGF expression in primary tumours did not predict metastases (odds ratio 1.01, 95% CI 1.0 to 1.02, *p* = 0.10). 

## 3. Discussion

Better knowledge of the characteristics of lymph node metastases of thyroid cancer is required to identify aggressive disease subsets that require additional treatments. Here, we report an increased expression of proNGF in thyroid cancer lymph node metastases, which correlated with expression in the primary tumour. However, we did not find that proNGF expression was able to predict the presence of these metastases, or that it correlated positively with tumour aggressiveness. 

A key novel aspect of this study was the assessment of proNGF expression in lymph node deposits of thyroid cancer. ProNGF expression has never previously been reported in the metastases of any cancer, despite significant study of its biological effects in primary tumours of breast and prostate cancers [8,12]. This study identifies that proNGF is a biological feature of lymph node metastases of thyroid cancer. More importantly, by studying paired primary tumours and metastases, we were able to show a clear correlation between the biological behaviour of proNGF expression in the primary tumour and the metastases. Interestingly, although there was strong positive correlation between proNGF expression in the primary tumour and its linked metastases, we found that the degree of proNGF expression in the metastases was inversely associated with nodes located in the lateral neck (a site of higher-risk involvement), and with male gender. 

This work provides important independent corroboration of the findings of a previous study of proNGF in thyroid cancer [11]. This current work confirms the pattern that the papillary subtype of thyroid cancer has higher levels of proNGF expression, as demonstrated previously. Further, this work confirms previous findings that proNGF expression in the primary tumour does not correlate with high risk clinical features. Table 2 shows that on multivariate analysis, there was no association between clinical or pathological risk-factors and proNGF expression. The observation that proNGF expression is lower in primary tumours harbouring high-risk features (seen on univariate analysis in Table 1) suggests that reduced proNGF expression could be a sign of early dedifferentiation. In the nervous system, proNGF/NGF expression is generally associated with neuronal differentiation [13], and it is plausible that in thyroid cancer, decreased expression of proNGF could similarly be associated with the dedifferentiation of cancer cells. 

An important aspect of the study design was the use of whole-slide analyses of cases with a complete clinico-pathological dataset, as this provided a more complete picture of protein expression across the tumour. We found that although proNGF expression was mostly uniform across primary tumours, a subset of tumours expressed proNGF in a peripheral pattern. It is possible that expression of proNGF may be induced by factors in the tumour microenvironment, such as oxygen, or that proNGF is a component of actively growing cells preferentially located at the periphery of solid tumours; however, further functional investigation is warranted to understand the meaning of preferentially-peripheral expression of proNGF. Additional strengths of this study are the use of automated techniques for immunohistochemistry staining, and digital quantification of protein expression. Manual techniques for immunohistochemistry and biomarker quantification have inherent risks of inter-slide variability and inter-observer bias. The automated techniques used in this study were appropriately trained and validated, and hence, the comparison of differences in staining intensities between tissues, as undertaken here, was more robust. 

The primary limitation of this study was the use of single modality immunohistochemistry to evaluate protein expression. However, risks of protein misidentification were minimised by using a well validated antibody against proNGF and appropriate controls. The specificity of proNGF targeting with the antibody used was confirmed against a monoclonal antibody in our previous study, as well as in Western blotting [11]. Minimal nonspecific staining was observed. An additional limitation was that this study included relatively few cases of follicular and anaplastic thyroid cancers and their metastases, and it is possible that these subgroups may exhibit different expression characteristics; as such, cautious interpretation is required. However, the large number of paired papillary thyroid cancer metastases included in this study allows for a robust interpretation of this cohort. 

On a broader perspective, proNGF may be of interest as a therapeutic target. In breast cancer, proNGF has been shown to stimulate cancer cell growth and dissemination [12,14], and in prostate cancer, proNGF expression is a driver of nerve infiltration in the tumour microenvironment [15] that is essential to tumour growth and dissemination [16]. The association between the presence of nerves, neurotrophic growth factor expression and cancer progression is an evolving field, with increasing recognition of the key role of nerve-derived factors in tumour progression [17]. Nerve-signalling has long been recognised as integral for tissue regeneration after injury, and it is hypothesised that similar mechanisms may promote neoplastic progression [17]. For example, denervation has been shown to reduce the incidence of gastric carcinoma [18] and basal cell carcinomas of the skin [19]. In animal models of prostate cancer, tumour progression in animal models is strongly inhibited after denervation [16,20]. Whether proNGF also contributes to thyroid cancer and the development of metastases should be experimentally explored both in vitro and in vivo. 

In conclusion, this study demonstrates proNGF expression in lymph node metastases of thyroid cancer which correlates with expression in the primary tumour, suggesting an underling biological link which warrants further investigation. 

## 4. Materials and Methods 

### 4.1. Patients and Samples 

The Hunter New England Local Health District Human Research Ethics Committee prospectively approved this study, which included a waiver of consent for access to archival material (approval number HNE HREC 16/04/20/5.13, approved April 2016). Electronic records were searched to find lymph node metastases of thyroid cancer specimens during 2007–2017, where the original primary tumour was also available (*n* = 56). Clinical parameters were extracted from the medical record, and an independent pathologist reviewed all histological samples to confirm the diagnosis. 

### 4.2. Immunohistochemistry

Sections (4 microns) were prepared from archival, formalin-fixed, paraffin-embedded tissue blocks immediately prior to staining. Immunohistochemistry was performed using the Ventana Discovery automated slide stainer (Roche Medical Systems, Tucson, AZ, USA). The primary antibody was an anti-rabbit polycloncal proNGF antibody (Ab9040, Merck Millipore, Darmstadt, Germany), which has been previously validated in our laboratory [11] and was reoptimised for automated tissue processing at a dilution of 1/350. Antigen-retrieval was performed using Ribo-CC solution, (pH 6, Ventana, Roche, North Ryde, NSW, Australia), with primary antibody incubation for 32 min, secondary anti-rabbit HQ for 16 min at 37 °C (Ventana, Roche, North Ryde, NSW, Australia) and tertiary anti-HQ (Ventana, Roche, North Ryde, NSW, Australia) for 16 min at 37 °C. Manual counterstaining was performed using Mayer’s haematoxylin for 10 s and Scott’s Tap Water for 30 s, followed by dehydration, clearing and mounting. Negative controls were prepared using nonspecific IgG isotype controls, and without the addition of primary antibody (Appendix A). 

### 4.3. Digital Quantification

To quantify biomarker expression, whole slides were digitised at 20x magnification using an Aperio AT2 scanner (Leica Biosystems, Victoria, Australia). Two operators (CR and TD) manually graded a training image library of 20 cancers to obtain consensus. QuPath whole-slide digital image analysis software (Queens University, Belfast, UK) [21] was then optimised to quantify maximum cytoplasmic DAB staining. Initially, a cell detection algorithm was optimised to detect nuclear and cytoplasmic regions of malignant cells. DAB threshold values were then determined from histogram analyses of the training library. Finally, the training library was used to build a cell detection classifier (to exclude fibroblasts, lymphocytes, erythrocytes and colloid) and optimise DAB thresholds to replicate the scoring in the training library. The classifier was then locked and batch-run on all slides. To minimise false positive cell detection and optimise processing time, tumour regions were manually defined. Areas of haemorrhage, dense stroma without malignant cells, dense lymphocytic infiltrate and folding artefacts were excluded. The intensity of DAB staining was quantified using a triple-threshold h-score for each tumour (calculated as the sum of 3× percentage of pixels with strong staining; 2× percentage of pixels with moderate staining and 1× percentage of pixels with weak staining). Examples of image analyses are shown in Appendix B. The quantification of intra-tumoural biomarker distribution in a peripheral or uniform pattern was performed by separately calculating h-score for the outer 1/3 and inner 1/3 of tumour. A peripheral > central pattern was defined as a peripheral: central h-score ratio >1, with an absolute difference in h-score >20 points. Uniformly positive was defined as total tumour h-score > 25 that did not meet the criteria for peripheral > central. Uniformly negative was defined as total tumour h-score ≤ 25, irrespective of the peripheral: central ratio. 

### 4.4. Statistical Analysis

A prior study showed that thyroid proNGF immunohistochemistry quantification by h-score was normally distributed with a standard deviation of 21 units [11]. To demonstrate a mean h-score difference of 25 units between groups, a minimum of 25 cases per group were required, with a power of 0.8 and two-sided alpha of 0.05.

We dichotomised age and tumour size based on thresholds in the 8th edition of the American Joint Cancer Committee TNM Staging System and tumour/node size, extra-thyroidal extension, node number and site on the 2015 American Thyroid Association guidelines [3]. We provide strata-level medians and interquartile ranges for h-scores across strata levels, compared using the Mann-Whitney test.

We modelled the association between h-scores and tumour types using linear quantile mixed models that include clinico-pathological covariates and account for within subject clustering. Using the paired tissue sections, we explored the association between the metastatic and primary tumour h-scores along with markers of aggressiveness using quantile regression. In instances where potentially influential points were identified, we excluded these observations and repeated the analyses to assess the sensitivity of parameter estimates. We provide point estimates, 95% confidence intervals and *p*-values for each of the model terms. All reference to significance assumes a 0.05 type-I error rate. All analyses were performed using Stata (version 14.1, Statacorp, Texas USA).

## Figures and Tables

**Table 1:** Expression of proNGF in primary thyroid cancers and metastases, quantified by h-score of immunohistochemical staining.

	ProNGF Quantification (h-score)					
	*n*	Primary Tumour	*p*-Value	*n*	Nodal Metastasis	*p*-Value
**Overall**	56	57 (42–84)		56	94 (50–147)	0.002 ^1^
**Age**			0.96			0.32
<55 years	34	58 (43–89)		34	95 (57–122)	
≥55 years	22	55 (32–77)		22	86 (46–123)	
**Sex**			0.20			0.85
Male	21	71 (46–100)		21	96 (46–148)	
Female	35	54 (40–71)		35	90 (51–146)	
**Histopathology**			0.36			0.11
Papillary	53	58 (43–89)		53	96 (51–148)	
Classical	27	54 (43–92)		27	106 (64–149)	
Follicular-variant	26	60 (46–89)		26	70 (49–130)	
Follicular	2	42 (23–60)		2	45 (30–60)	
Anaplastic	1	32		1	67	
**Tumour size**			0.02			
Primary <4 cm	48	62 (45–96)				
Primary ≥4 cm	8	38 (25–55)				
Total lymph node <3 cm				44	105 (57–149)	0.02
Total lymph node ≥3 cm				12	59 (44–64)	
Metastasis within node <2 mm				10	148 (94–175)	0.04
Metastasis within node ≥2 mm				46	82 (49–123)	
**Vascular invasion**			0.32			
Absent	35	56 (43–100)				
Present	21	58 (43–68)				
**Extra-thyroidal extension**			0.43			
Absent	19	56 (45–106)				
Present	37	58 (40–75)				
**AJCC TNM 8 Stage**			0.005			
I and II	46	63 (45–100)				
III and IV	10	38 (23–54)				
**Nodal metastases (location)**						0.004
Central neck only (N1a)				33	109 (64–152)	
Central + lateral neck (N1b)				23	60 (40–96)	
**Timing of metastases**						0.11
At time of primary tumour				49	99 (56–148)	
>6 months from primary				7	57 (46–96)	

The h-score is a digital quantification of immunohistochemistry intensity, with values ranging between 0 and 300, where higher values represent greater protein expression. Data are median (IQR), compared using the Mann-Whitney test. ^1^ Comparison between Primary and Metastases. Other comparisons are within column.

**Table 2:** Multivariate analysis of association between h-score in primary or metastases and clinical/pathological parameters. Parameter estimates for linear quantile mixed models of association between median proNGF h-score and clinical/pathological parameters are presented. Separate models were fit for (**A**) primary tumours and (**B**) Lymph node metastasis. The estimate shows the difference in h-score associated with the parameter.

Parameter	Estimate (95% CI)	*p*-Value
**(A) Primary Tumour:**		
Age (>= 55 years)	−6.1 (−35.9 to 23.7)	0.7
Sex (male)	−2.7 (−27.3 to 21.8)	0.8
Size (>= 4 cm)	−9.1 (−28.8 to 10.6)	0.4
Extra-thyroidal extension	−14 (−29.0 to 1.0)	0.067
Vascular Invasion	−11.6 (−36.4 to 13.2)	0.4
Multi-focal	−1.7 (−19.1 to 15.7)	0.8
**(B) Lymph node metastasis**		
Age (>= 55 years)	−13.8 (−32.5 to 4.9)	0.1
Sex (male)	−34.8 (−59.9 to −9.6)	0.008
Size (>= 2 mm)	−29.1 (−65.34 to 7.2)	0.1
Location (lateral neck site, N1b)	−47.1 (−73.8 to −20.5)	0.001
Timing (> 6 months post primary)	−23.7 (−52 to 4.6)	0.1

**Figure A1:**
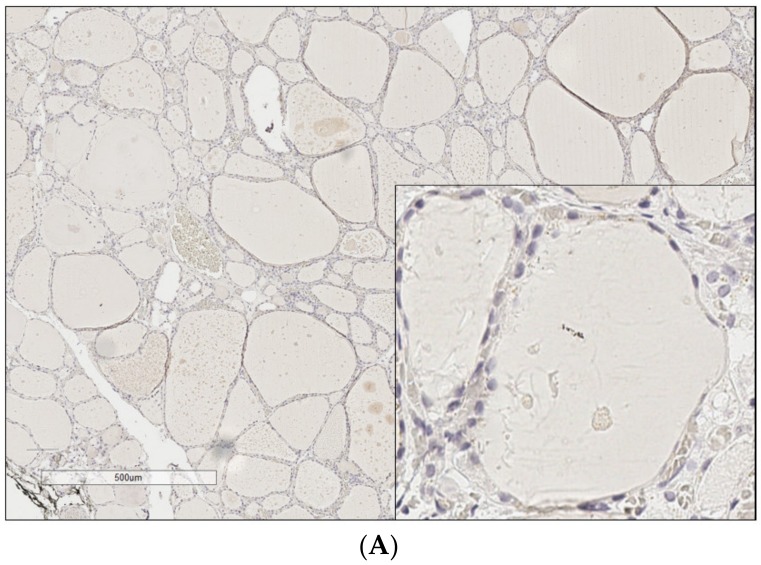

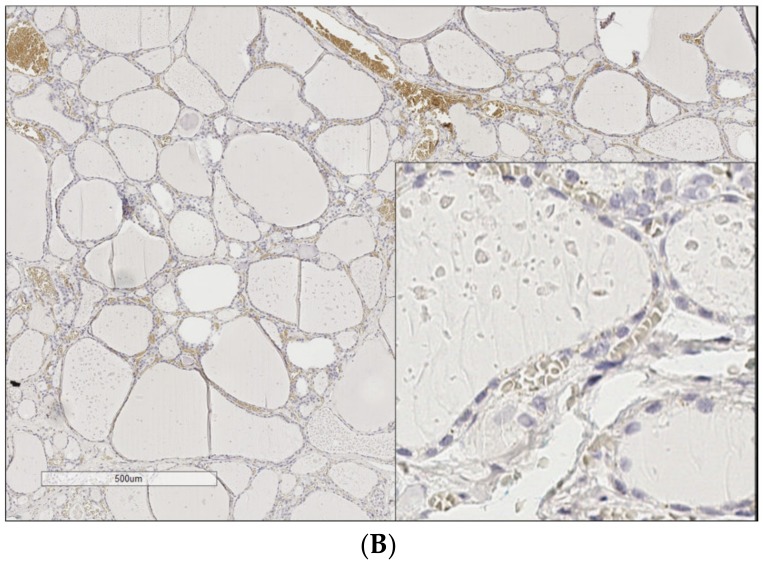
Negative Controls for proNGF staining. (**A**) IgG isotype control. Nonspecific IgG was added in place of the primary antibody. (**B**) Without primary antibody. Nonspecific brown staining is of red blood cells. For positive controls, see Faulkner et al. (2016) [5].

**Figure A2:**
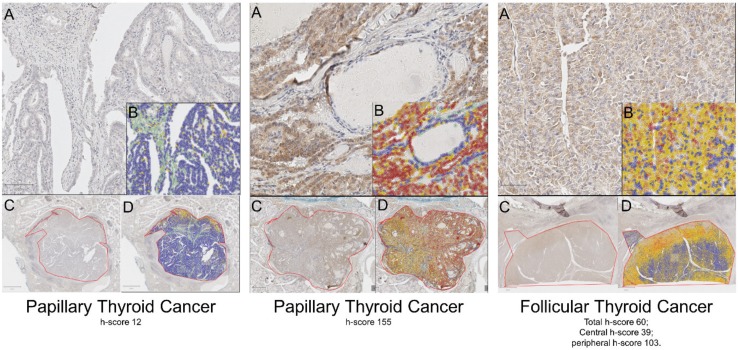
Examples of QuPath grading of cytoplasmic DAB intensity of three separate tumour cases. Each case has 4 separate panels presented (**A**–**D**). (**A**): High magnification (20×) image of immunohistochemistry staining of tumour. (**B**): QuPath markup of same region showing cell detection and classification. (**C**) Low magnification (4×) image of tumour, demarcated in red. (**D**): Low magnification image of tumour with QuPath markup. Markup: Green (stroma), Purple (immune cells), Blue (negative tumour cells).

## Abbreviations

AJCC	American Joint Committee on Cancer
DAB	3′3′ diaminobenzidine
ProNGF	Precursor for nerve growth factor
TNM	Tumour, Nodes, Metastases Staging System
